# Supplementary material for: KDM4 Regulates the Glycolysis of Hemocytes in the Immune Priming of Eriocheir sinensis
Source: Int J Mol Sci. 2024 Dec 7;25(23):13174. doi: 10.3390/ijms252313174 (PMC11642315; doi:10.3390/ijms252313174)
Supplement: Supplementary file 1 [file ijms-25-13174-s001.zip › ijms-3295087-supplementary.pdf]

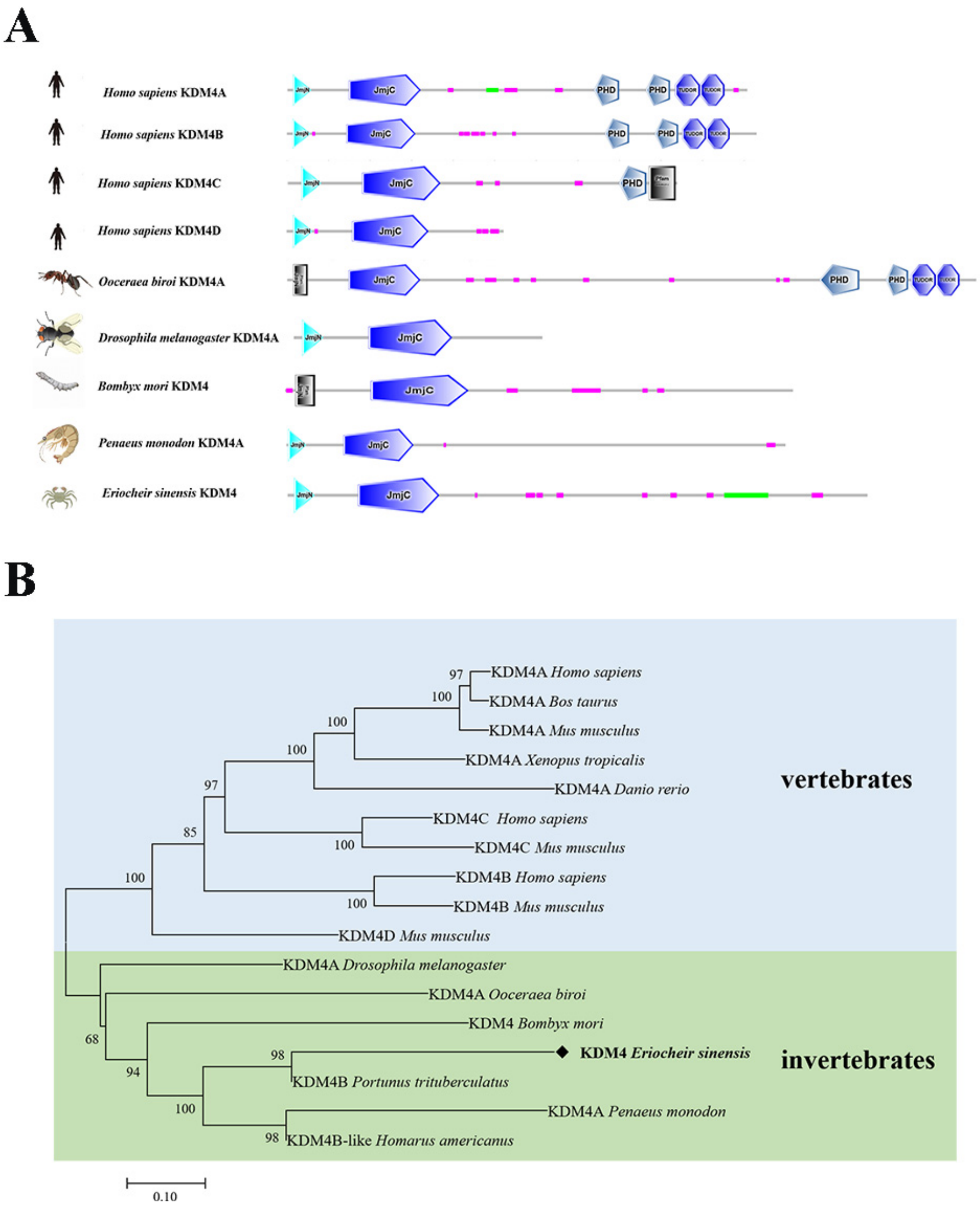

Figure S1. The domain characters and phylogenetic relationships of KDM4 sequences

(A) Protein domains of KDM4 from different species. (B) Evolutionary tree of KDM4s constructed by MEGA 6.0 program with neighbor-joining (NJ) algorithm. The GenBank accession numbers were as follow: NP 055478.2 *Homo sapiens,* KDM4A; NP 001193245.1 *Bos taurus*, KDM4A; NP 001413186.1 *Mus musculus*, KDM4A; NP 001107498.1 *Xenopus tropicalis*, KDM4A; XP 005165745.1 *Danio rerio*, KDM4A; NP

001140168.1 *Homo sapiens,* KDM4C; NP 001343490.1 *Mus musculus*, KDM4C; NP 001398077.1 *Homo sapiens*, KDM4B; NP 742144.1 *Mus musculus*, KDM4B; NP 001397435.1 *Mus musculus*, KDM4D; sp|Q9V333.1| *Drosophila melanogaster*, KDM4A; XP 011337810.1 *Ooceraea biroi*, KDM4A; NP 001243917.1 *Bombyx mori*, KDM4; XP 050717641.1 *Eriocheir sinensis*, KDM4; MPC29560.1 *Portunus trituberculatus*, KDM4B; XP 037797354.1 *Penaeus monodon*, KDM4A; KAG7159756.1 *Homarus americanus*, KDM4B-like.

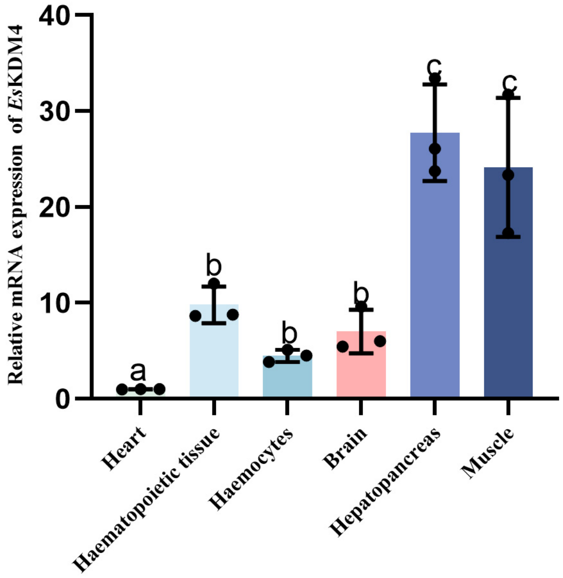

Figure S2. The mRNA expression level of *Es*KDM4 in different tissues

Comparison of the expression level of *Es*KDM4 mRNA (relative to *Es*β-actin) among different tissues was normalized to heart. Hep: hepatopancrease; Hea: heart; HPT: Haematopoietic tissue; Bra: brain; Mus: muscle; Hae: haemocyte. Vertical bars represent the mean ± S.D. (N=3) for each tissue. The different letters (a, b, c etc.) indicated significant differences ($p < 0.05$, ANOVA).

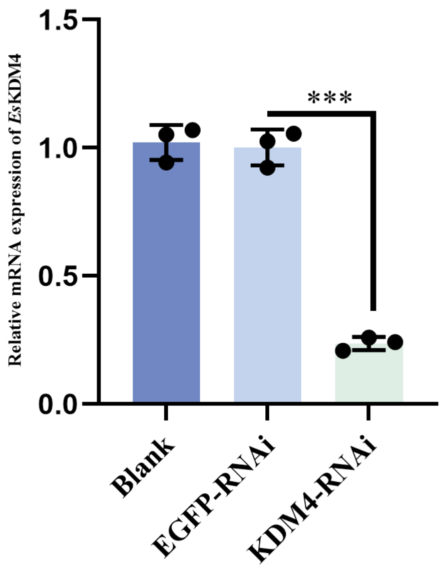

Figure S3. The efficiency of siRNA-KDM4 in haemocytes was analyzed by qPCR. Crabs were injected with siRNA-KDM4 or siRNA-EGFP, with siRNA-EGFP serving as a control. In the Blank group, crabs were not received any treatment. At 12 h after the injection of siRNA-KDM4, the haemocytes were collected to verify efficiency of siRNA-KDM4 (N=3).
